# Expert consensus on orthodontic treatment of protrusive facial deformities

**DOI:** 10.1038/s41368-024-00338-4

**Published:** 2025-02-01

**Authors:** Jie Pan, Yun Lu, Anqi Liu, Xuedong Wang, Yu Wang, Shiqiang Gong, Bing Fang, Hong He, Yuxing Bai, Lin Wang, Zuolin Jin, Weiran Li, Lili Chen, Min Hu, Jinlin Song, Yang Cao, Jun Wang, Jin Fang, Jiejun Shi, Yuxia Hou, Xudong Wang, Jing Mao, Chenchen Zhou, Yan Liu, Yuehua Liu

**Affiliations:** 1 Department of Orthodontics, Shanghai Stomatological Hospital & School of Stomatology &Shanghai Key Laboratory of Craniomaxillofacial Development and Diseases, Fudan University, Shanghai, China; 2 Department of Orthodontics, Shanghai Ninth People’s hospital, school of medicine, Shanghai Jiao Tong university, Shanghai, China; 3 Department of Orthodontics, Peking University School and Hospital of Stomatology & National Center for Stomatology & National Clinical Research Center for Oral Disease, Beijing, China; 4 Center of Stomatology, Tongji Hospital & School of Stomatology, Tongji Medical College, Huazhong University of Science and Technology & Hubei Province Key Laboratory of Oral and Maxillofacial Development and Regeneration, Wuhan, China; 5 Orthodontic Department, Stomatological School, Wuhan University, Wuhan, China; 6 Department of Orthodontics, Beijing Stomatological Hospital, School of Stomatology, Capital Medical University, Beijing, China; 7 College of Stomatology, Nanjing Medical University, Nanjing, China; 8 Department of Orthodontics, School of Stomatology, The fourth military medical university, Xi’an, China; 9 Department of Orthodontics, School and Hospital of Stomatology, Jilin University, Changchun, China; 10 College of Stomatology & Chongqing Key Laboratory of Oral Diseases & Chongqing Municipal Key Laboratory of Oral Biomedical Engineering of Higher Education, Chongqing Medical University, Chongqing, China; 11 Hospital of Stomatology, Guanghua School of Stomatology, Sun Yat-sen University, Guangdong Provincial Key Laboratory of Stomatology, Guangzhou, China; 12 State Key Laboratory of Oral Diseases & National Clinical Research Center for Oral Diseases & Department of Orthodontics, West China Hospital of Stomatology, Sichuan University, Chengdu, China; 13 Department of Orthodontics, Zhejiang University Affiliated Stomatological Hospital, Hangzhou, China; 14 Department of Orthodontics, College of Stomatology, Xi’an Jiaotong University, Xi’an, China; 15 Department of Oral and Cranio-maxillofacial Surgery, Shanghai Ninth People’s Hospital, Shanghai Jiao Tong University School of Medicine; College of Stomatology, Shanghai Jiao Tong University; National Center for Stomatology; National Clinical Research Center for Oral Diseases; Shanghai Key Laboratory of Stomatology; Shanghai Research Institute of Stomatology; Research Unit of Oral and Maxillofacial Regenerative Medicine, Chinese Academy of Medical Sciences, Shanghai, China

**Keywords:** Craniofacial orthodontics, Malocclusion

## Abstract

Protrusive facial deformities, characterized by the forward displacement of the teeth and/or jaws beyond the normal range, affect a considerable portion of the population. The manifestations and morphological mechanisms of protrusive facial deformities are complex and diverse, requiring orthodontists to possess a high level of theoretical knowledge and practical experience in the relevant orthodontic field. To further optimize the correction of protrusive facial deformities, this consensus proposes that the morphological mechanisms and diagnosis of protrusive facial deformities should be analyzed and judged from multiple dimensions and factors to accurately formulate treatment plans. It emphasizes the use of orthodontic strategies, including jaw growth modification, tooth extraction or non-extraction for anterior teeth retraction, and maxillofacial vertical control. These strategies aim to reduce anterior teeth and lip protrusion, increase chin prominence, harmonize nasolabial and chin-lip relationships, and improve the facial profile of patients with protrusive facial deformities. For severe skeletal protrusive facial deformities, orthodontic-orthognathic combined treatment may be suggested. This consensus summarizes the theoretical knowledge and clinical experience of numerous renowned oral experts nationwide, offering reference strategies for the correction of protrusive facial deformities.

## Introduction

The protrusive facial deformity typically refers to the malocclusion where the upper and lower lips are protruded relative to the facial profile.^1–3^ It includes skeletal Class I protrusion, characterized by the protruding upper and lower incisors, with or without forward-positioned jaws, and a mostly neutral molar relationship.^4,5^ The more common facial deformity in China is skeletal Class II protrusion, which refers to a malocclusion characterized by a discrepancy in the three-dimensional relationship between the upper and lower jaws, accompanied by dental compensation, with distal or neutral molar relationships.^6–8^ Among various misalignments in the sagittal, transverse, and vertical dimensions, the thickness of soft tissues can also impact the morphology of hard tissues.^9^ Compensatory interactions among the perioral muscles, teeth, and jaws may present completely different soft tissue profiles. Failure to consider the comprehensive coordination of teeth, jaws, and soft tissues, as well as the complicated mechanisms of malocclusion, during the formulation of orthodontic plans can often lead to incorrect assessments by orthodontists regarding treatment goals, difficulty levels, and outcomes. Therefore, a multidimensional analysis and judgment of the etiologic mechanisms of protrusive facial deformity are necessary to develop correct treatment plans.^10,11^

The formulation of orthodontic treatment plans normally involves consideration of multiple dimensions and comprehensive elements. Multiple dimensions refer to the traditional three-dimensional concept, including the sagittal, vertical, and transverse dimensions and maturity of growth and development.^12^ Multi-elements include teeth alignment, jaw relationship, facial contour, periodontal condition, upper airway patency, temporomandibular joints, and perioral muscle balance.^13–15^ The treatment plan, based on multidimensional analysis, differs from traditional orthodontic approaches that primarily focus on diagnosis and treatment concepts limited to teeth, jaws, and facial profiles. This consensus underscores the utilization of various orthodontic strategies, such as mandible advancement, retraction of anterior teeth, and maxillofacial vertical control.^16,17^ These strategies aim to reduce anterior teeth and lip protrusion, increase chin prominence, harmonize nasolabial and chin-lip relationships, and improve the facial profile of patients with protrusive facial deformities. In cases of severe skeletal protrusive facial deformities, a combination of orthodontic and orthognathic treatments may be recommended.^18–20^

## The etiological mechanisms and clinical manifestations of protrusive facial deformities

Protrusive facial deformities are characterized by the forward position of the lips relative to the facial profile, assessed through the position of three critical anatomical landmarks, including the glabella, subnasale, and pogonion.^21^ Diagnosed by focusing on soft tissue morphology, these deformities include a variety of complex maxillofacial abnormalities with compromised soft-tissue contours, closely related to the upper airway, temporomandibular joint, and perioral muscle balance.^22^ The “functional matrix theory” of growth posits that facial growth is a response to functional needs and neurotrophic influences.^23,24^ Effective lip competence and nasal breathing are essential for the synchronized development of maxillofacial elements, as normal respiratory airflow during these activities stimulates the development of relevant anatomical areas, thereby enhancing craniofacial structures.^25^ Protrusive facial deformities are often accompanied by decreased muscle tone, which affects the morphology of the underlying bone structures and prompts compensatory adjustment among the lips, teeth, and jaws, ultimately resulting in an imbalanced soft tissue contour.^26^ These deformities can also be caused by obstructive airway conditions exacerbating structural defects manifested by narrowed dental arches, elevated palates, hypoplasia of the face and nose, and excessive divergent skeletal Class II deformities known as “adenoid facies”.^27,28^

**Fig. 1:**
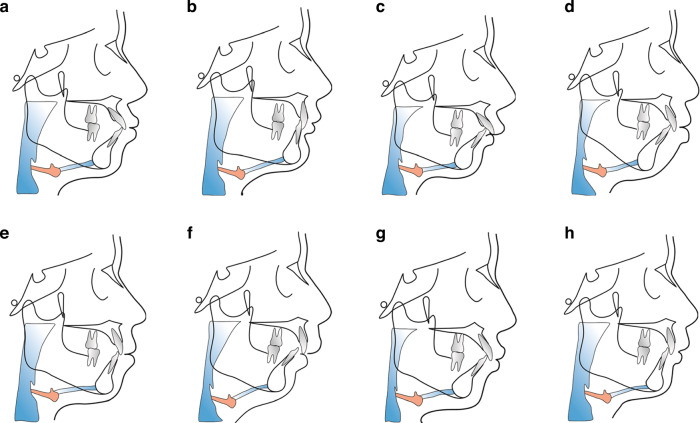
Schematic diagram of different protrusive facial deformities. **a** Normal maxilla and mandible with upper and lower incisors proclination, small nasolabial angle, and prominent lips. **b**–**d** Retruded mandibular and normal maxilla relative to the cranial base, which is the main type of facial protrusive deformities in Chinese population. This type can be divided into horizontal growth pattern (**b**), average growth pattern (**c**), and vertical growth pattern (**d**). **e** Bimaxillary protrusive deformities with mentalis strain on lip closure. **f** Maxillary protrusion and mandibular retrusion, accompanied by compensatory lingual inclination of upper incisors and proclination of lower incisors. **g** Insufficient maxilla with severe mandibular retrusion and proclined upper and lower incisors. **h** Protruded maxilla and normal mandible, accompanied by lingually inclined upper incisors and proclination of lower incisors

The morphological mechanisms and clinical manifestations of protrusive facial deformities are diverse and complex. The simple form typically presents proclination of the upper and lower incisors, and a Class I molar relationship, which might be accompanied by maxillary and mandibular protrusion. Severe protrusive facial deformities may be accompanied by sagittal discrepancy between the jaws, excessive vertical growth, and abnormal molar relationships, which may result in dentofacial dysfunction.^29^ The complex etiology and pathogenesis of these deformities are diverse, resulting in a wide range of craniofacial morphologies, that need to be classified according to specific skeletal sagittal and vertical determinants (Fig. 1).

Epidemiological data reveal a high prevalence of protrusive facial deformities in China, typically characterized by normal maxilla and insufficient mandible.^30^ Given the multifactorial etiology and complex clinical manifestation of these deformities, a holistic assessment is essential to establish an accurate diagnosis and treatment plan, which should be followed by the multidimensional and total-element analysis strategies, to achieve the esthetic, functional and stable goals of orthodontic treatment.

## Principals of orthodontic treatment for protrusive facial deformities

Given the intricate etiology and varied clinical manifestations, refining the diagnostic strategy for protrusive facial deformities characterized by protrusion is crucial for improving both orthodontic outcomes and patient satisfaction. Typically, orthodontic strategies include multidimensional assessments and total-element considerations.

Multidimensional analysis refers to the comprehensive evaluation of sagittal, vertical and horizontal dimensions as well as growth potential in diagnosing and decision-making process of orthodontic cases.^31^ Complex protrusive facial deformities often involve abnormalities in multiple dimensions.^32^ Patients with hyperdivergent malocclusion exhibit excessive vertical facial growth and rotation of the mandible, resulting in an increased mandibular plane angle.^33^ The lower lip appears more prominent relative to the chin due to the clockwise rotation of the mandible in these patients, indicating that excessive retraction of incisors is not acceptable, resulting in excessive flattening of the lips. A vertical control strategy may be used to reduce the vertical height and alleviate the sagittal discrepancy.^34^ Due to favorable chin, hypodivergent skeletal Class II patients can obtain good profile by retraction of incisors to reduce lip prominence.^35^ Therefore, the extraction plan should be carefully selected to avoid excessive retraction of the incisors to form a concave profile.^36^

The normal width of the upper arch is the key to guide the sagittal growth of mandible.^37^ In children and adolescents, the narrow upper arch will limit the forward growth of the mandible, which causes the mandible to maintain the retrusive position, resulting in skeletal Class II malocclusions.^38^ The transverse discrepancy is closely related to facial esthetics.^39^ For patients with normal to large facial width, over-intruding the posterior teeth and counterclockwise rotation of the mandible are not recommended to avoid the deterioration of the facial width-to-length ratio.

Based on the three-dimensional analysis in sagittal, vertical and horizontal dimensions, we emphasize the fourth dimension-growth and development. As a classic concept in orthodontics, growth and development is limited to children and adolescents by many orthodontists, which is not comprehensive. The growth and development we emphasize refer to the changes of dentofacial hard and soft tissues throughout the life cycle and the decisive role of genetic factors on growth patterns.^40^

**Fig. 2:**
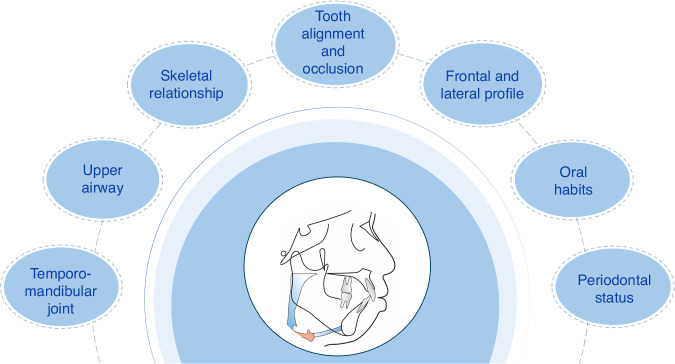
Schematic diagram of total-element analysis

The etiology of malocclusion is multifaceted, including teeth alignment, jaw relationship, facial contour, periodontal condition, upper airway patency, temporomandibular joints, and muscle balance. Failure to consider the clinical manifestations and the malocclusion formation mechanism may lead to incorrect prediction of the goal, difficulty index, strategy, and efficacy of orthodontic treatment. Therefore, the optimal treatment plan should be formulated based on the total-element diagnosis and comprehensive analysis (Fig. 2).

### Tooth alignment and occlusion

Tooth alignment involves a variety of elements, including mesiodistal angulation, labiolingual angulation, rotation, crowding, labiolingual malposition, and arch form.^41^ The goal of orthodontic treatment is to achieve a combination of the above elements with good occlusal function, and the roots should be centered within the alveolar bone to maintain periodontal health.^42^ In patients treated with camouflage orthodontics, the root in the alveolar bone can be moderately displaced from the center and exhibit compensatory inclination within a safe range, achieving ideal tooth alignment and occlusion.^43^

In addition to the above elements, individual patients’ soft tissue characteristics should also be considered. For different vertical skeletal patterns and different face shapes, the arrangement of teeth should be considered individually.^44^ The dental arch should match the face shape.^45^ The appropriate width of the dental arch contributes to a coordinated buccal corridor. Otherwise, it can negatively impact smile esthetics.^46^

### Skeletal relationship

The skeletal relationship includes sagittal and vertical skeletal patterns.^47^ In addition to considering the relative position of the maxilla and mandible, the absolute sagittal jaw positions relative to the cranial base should also be considered in the diagnosis of a patient’s sagittal skeletal pattern, which is crucial for determining the target positions in orthodontic treatment planning.^48^ The impact of the vertical skeletal pattern on the sagittal pattern should also be considered in diagnosis and treatment plan design.^49^ Hyperdivergent skeletal pattern aggravates skeletal Class II malocclusion, while hypodivergent skeletal pattern aggravates skeletal Class III malocclusion.^50^ The jaw positional relationship is an important part of the dentofacial complex system, which should be integrated with soft tissue analysis to formulate the most appropriate treatment plans for patients presenting with protrusive facial deformities.

### Frontal and lateral profile

Both the goals and limitations of modern orthodontic and orthognathic treatment are determined not only by the teeth and bones but also by the soft tissues of the face.^51^ The “soft tissue paradigm” advocated by modern orthodontics is to calculate the target position of incisors according to the soft tissue esthetics, so as to determine the details of treatment design such as the required space, the pattern of extraction, and the anchorage design.^52^ When making treatment plans, orthodontists should pay more attention to the patient’s frontal and lateral profile, and strive to make the treatment plan consistent with or close to the patient’s subjective esthetic expectations.^53^ Attention should also be paid to the adverse changes in profile caused by orthodontic treatment, particularly in patients with high zygomatic bone, sunken cheeks, etc. ^45,54^

### Other elements

The upper airway is closely related to health and life, and it is one of the key elements to be considered in the diagnosis and treatment of malocclusion.^55^ Following the retraction of incisors, the velopharyngeal, glossopharyngeal, and hypopharyngeal airway may become narrower.^56^ The target position of incisors should not only meet the needs of facial esthetics but also take into account the effect of incisor retraction on the size of the upper airway.^57^ For patients with upper airway stenosis, the amount of incisor retraction should be strictly controlled to maintain the inherent oral space and normal nasal respiratory function. If necessary, bimaxillary advancement surgery should be combined to correct the protrusion and increase the airway volume.^58^

Oral habits, such as sucking habit, abnormal tongue position, and tongue thrust swallowing, can break the balance of the internal and external strength of the jaw and arch, leading to malocclusion.^59^ Within the stomatognathic system, muscles often play a dominant role over bones. For patients exhibiting oral habits, orthodontists should ensure that changes in teeth, arches, and jaws are coordinated with muscle function during treatment. Only by removing oral habits and achieving normal perioral muscle function can ensure the long-term stability of orthodontic treatment.^60^

Temporomandibular disorders (TMD) are primarily characterized by joint pain, joint noise, and mandibular movement disorders.^61^ It is necessary to recognize the complexity of the etiology and pathophysiological mechanism of TMD and its impact on the stability of mandibular position and occlusion.^62,63^ A high prevalence of TMD in skeletal class II patients referred for orthognathic surgery, especially in those with a pronounced overjet and high mandibular plane angle. A solid cusp-fossa relationship of the teeth should be established during orthodontic treatment, which is an important factor for the long-term stability of tooth alignment and occlusion.^64^

The periodontal status, including gingival texture, periodontal pocket depth, tooth mobility, gingival recession, and alveolar bone level, should be evaluated before and during the orthodontic treatment.^65^ CBCT can also be used to evaluate the alveolar ridge height, alveolar bone thickness, alveolar ridge integrity (bone dehiscence and bone fenestration), and the relationship between root and bone.^66^ For patients with periodontal disease, the orthodontic treatment plan should be adjusted, the range of tooth movement should be reduced, and the communication between orthodontists and periodontists during the whole treatment process should be emphasized.^67^

## Orthodontic therapies for protrusive facial deformities

### Jaw growth modification for protrusive facial deformities

Mandibular functional treatment is a process in where orthodontists reposition the mandible after comprehensive evaluation of the stomatognathic system.^68^ Based on the growth potential of adolescents, the direction and extent of jaw growth should be effectively guided so as to alleviate the severity of skeletal malocclusions and improve the soft tissue contours.^69^ Orthodontic functional treatment should be initiated during the mixed dentition and early permanent dentition stages to take advantage of growth potential.^70^ The growth guidance of the mandible should be three-dimensional, involving the sagittal advancement accompanied by vertical and transverse adjustment.^71,72^ In particular, vertical control could be emphasized in this process of mandibular advancement.^73,74^

#### Adolescents with a low mandibular plane angle

Adolescents with a low mandibular plane angle generally exhibit well-developed mandibular symphysis but may present with reduced prominence of soft tissue chin due to mandibular deficiency. Mandibular advancement devices (MADs), such as the activator and the twin-block can be used for mandibular advancement treatment.^75^ During the process, the eruption and reconstruction of the posterior teeth should be guided, to improve the growth pattern by increasing the facial height of the lower one-third and relieve the deep overbite of anterior teeth, which is beneficial for individuals with a short face pattern.

#### Adolescents with a normal mandibular plane angle

The treatment approach for adolescents with normal-angle dentofacial profiles is similar to that for those with low-angle deformities. Adolescents with significant sagittal jaw discrepancy can be treated with MADs combined with extraction orthodontic treatment. However, in the case of normal-angle adolescents, it is essential to maintain the lower facial height during mandibular advancement to prevent the elongation of molars and a clockwise rotation of the mandible, both of which could diminish chin prominence.^76^

#### Adolescents with a high mandibular plane angle

The normal counterclockwise growth pattern of the mandible is transitioned to a clockwise trend in the hyperdivergent mandibular retrusion.^77^ Orthodontic treatment for adolescents exhibiting a long-face growth pattern is challenging.^78^ Due to the forward and inferior inclination of the occlusal plane, traditional MADs promote the saggital mandibular growth while adversely increasing the vertical dimension, resulting in unsatisfactory advancement of the mandible.^79^ The treatment of hyperdivergent skeletal Class II malocclusion is controversial.^74,80^ Extraction treatment can retract the anterior teeth and improve lip prominence to a certain extent but has little effect on improving chin projection.^35^ Some scholars believe that Herbst or Twin-Block appliances with a vertical control strategy are recommended for hyperdivergent skeletal Class II malocclusion, with attention to leveling of the upper anterior occlusal plane.^81–84^ The above approaches can avoid posterior teeth over-eruption and help to maintain the lower facial height during treatment.^85^ It should be emphasized that functional orthodontic treatment is not suitable for adolescents with a high mandibular plane angle, large lower facial height, and severe mentalis strain during lip closure.

### Anterior teeth retraction for protrusive facial deformities

The pathogenesis of protrusive facial deformities manifests in two main forms: dental protrusion and Class II skeletal malocclusion. In all these cases, soft tissue features typically include upper lip protrusion and a compromised nasolabial relationship. The sagittal position and torque of the upper and lower anterior teeth influence the prominence of the upper and lower lip.^86^ Adjusting nasolabial and mentolabial appearance can enhance lateral appearance, contributing to overall facial harmony.^2^

#### Routine teeth extraction and anterior teeth retraction

For cases of dental-facial protrusive deformities, teeth extraction is an effective treatment.^87^ For adolescent and adult skeletal malocclusion patients who have poor functional correction, and do not opt for orthognathic surgery, their options are limited to compensatory measures involving tooth and alveolar bone adjustments to address sagittal jaw discrepancies.^88^ This typically involves orthodontic extraction camouflage treatment, which can often compromise both appearance and stability.^43^

##### Assessment of soft tissue profile

Maximizing the use of extraction space and precisely controlling the sagittal position and torque of the anterior teeth are crucial for ensuring treatment efficacy.^89^ To achieve a favorable retraction effect, ample space is essential for the anterior teeth to retract adequately. This is because changes in lip and tooth protrusion are disproportionate.^90^ Typically, a 1 mm retraction of incisors results in an ~0.6 mm decrease in lip protrusion.^91,92^ Effective control of root retraction of the upper incisors is pivotal for reducing maxillary basal bone prominence.^93^

##### Teeth extraction mode

For the treatment of protrusive facial deformities, choosing the right extraction mode depends on the severity of the protrusion. Extraction of anterior teeth helps alleviate anterior crowding and protrusion, and posterior tooth extraction helps to alleviate posterior crowding and control vertical dimensions. For adolescents and adults who undergo extraction, a common approach is to extract four first or second premolars or the extraction of maxillary first premolars and mandibular second premolars.^94,95^ Additionally, in cases of open bite, extracting second premolars can help correct occlusal issues and facilitate better vertical control.

#### Dentition distalization without premolar tooth extraction

Orthodontists should be cautious when choosing to extract teeth.^96^ For example, patients with periodontitis and mild protrusion may be treated without tooth extraction. Interproximal enamel reduction, when necessary, can alleviate mild crowding and minimize the occurrence of “black triangles”.^96^ Orthodontic appliances like implant anchorage, the Pendulum appliance, the Frog appliance, and the extraoral arch can be used to achieve comprehensive distal movement of the teeth, correcting protrusion and deep overjet.^97–99^ Implant anchorage, strategically placed in the subzygomatic ridge area, enables distal movement of the upper teeth without causing root interference.^100^

### Vertical control strategies for protrusive facial deformities

Previous studies and clinical observations have established that orthodontic treatment lacking vertical control could lead to tooth elongation and an increase in facial height.^34,40,51^ While this may benefit individuals with low-angle facial deformities, it often worsens the facial profile in individuals with high-angle facial protrusion. Vertical control can result in a counterclockwise rotation of the mandible, but this is still a contentious issue.^101,102^ Our perspective is that vertical control strategies include the maintenance type and the mandibular counterclockwise rotation type, with the MP-SN angle being a key index.

The maintenance type of vertical control alone typically does not suffice to improve facial profile. However, when combined with tooth extraction treatment, it can significantly improve the profile of individuals with protrusive facial deformities. In contrast, the other type of vertical control involves mandibular counterclockwise rotation achieved by reducing the height of the dental arches using various techniques.^103^ Importantly, the mandibular counterclockwise rotation type of vertical control represents a potentially independent and effective approach to ameliorating protrusion deformities.

#### Orthodontic mechanisms of vertical control through counterclockwise rotation of the mandible

##### The mechanisms of vertical control in adolescents

Adolescents with facial protrusion are typically accompanied by a retrusive mandible. When employing orthodontic appliances, such as Twin-Block, Activator, Frankle II, and inclined guide plates in adolescents with high-angle protrusion, the potential risk of increasing vertical height must be carefully considered.^104^ In these cases, supplementary techniques such as J-hook appliances, auxiliary archwires, and implant anchorage can be utilized to control the vertical facial height and level the occlusal plane. This approach aims to induce counterclockwise rotation effect in mandibular growth and promote development of a Class I skeletal facial type.^105–107^

##### The mechanisms involved in mandibular counterclockwise rotation in adults

In adults, regardless of whether the patient presents with an anterior open bite or deep overbite, any occlusal contact during closure impedes the counterclockwise rotation of the mandible. The center of resistance for the mandible’s counterclockwise rotation is located in the condylar region. Through “compressing” the vertical height of the upper and lower tooth-alveolar bone complex, the mandible rotates forward and upward, driven by the action of the jaw-closing muscles. Generally, the vertical control technique involving mandibular counterclockwise rotation primarily focuses on intruding the teeth, reducing vertical dimension, and creating space for mandible to rotate, which contributes to the overall improvement of facial esthetics and occlusal function.^108^

#### Indications for mandibular counterclockwise rotation type of vertical control

##### Personality factors of patients

Tooth intrusion poses a risk of root resorption,^109^ necessitates active patient cooperation and understanding of potential risks. Patients with neurotic personality traits, such as anxiety and distrust, require strengthened doctor-patient communication and careful consideration of treatment plans.

##### Consideration of the vertical height of the upper and lower arches

The vertical height of the upper and lower arches varies among individuals with high-angle facial deformities. Parameters, such as U1-PP (vertical height of upper anterior teeth), U6-PP (vertical height of upper posterior teeth), L1-MP (vertical height of lower anterior teeth), and L6-MP (vertical height of lower posterior teeth), should be evaluated clinically and using imaging. These values inform the selection of the intrusion site and the design mode of mandibular counterclockwise rotation type of vertical control.

##### Soft-tissue contour factors

The patient’s soft tissue profile influences the selection of the intrusion site. If a patient’s smile shows insufficient teeth exposure due to excessive soft tissue length of the upper lip, methods involving intrusion of the upper anterior teeth may not be suitable.

#### Vertical control strategies for counterclockwise rotation of the mandible

**Fig. 3:**
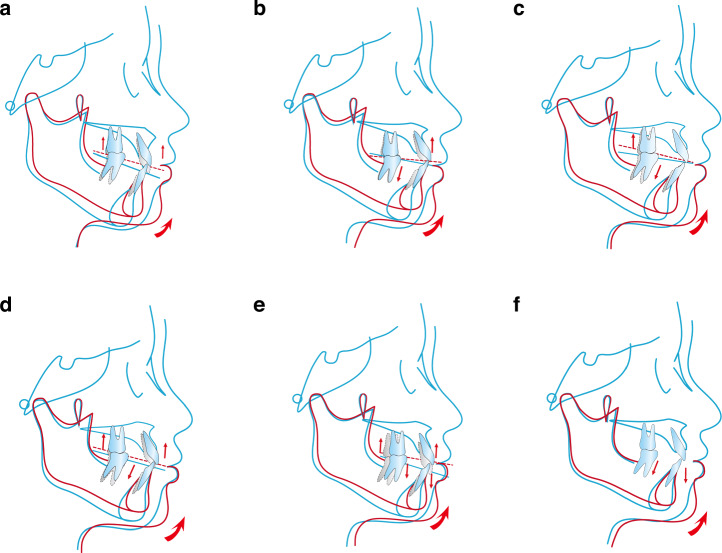
Schematic diagram of vertical control strategies for the inverse rotation of the mandible. **a** Gummy smile with vertical overdevelopment of the upper arch. **b** Gummy smile with overdeveloped upper anterior and lower posterior teeth. **c** No gummy smile with open bite anterior teeth and vertically overdeveloped upper and lower posterior teeth. **d** Gummy smile with open bite anterior teeth and vertical overdevelopment of upper and lower posterior teeth. **e** Gummy smile with vertically overdeveloped upper and lower teeth. **f** No gummy smile with normal upper arch and vertically overdeveloped lower arch

The strategy involving mandibular counterclockwise rotation in individuals with high-angle facial deformities requires careful consideration of individual variations in facial contour, as well as clinical and radiographic parameters of the dental arches. Based on these factors, a personalized combination of intrusions can be selected for different manifestations of high-angle facial deformities. It should be noted that the counterclockwise rotation of the mandible is not based on the premise of the counterclockwise rotation of the occlusal plane.^110^ The following is a classification (Fig. 3):

- Gummy smile with vertical overdevelopment of upper arch: Intrusion of upper anterior and posterior teeth, upward displacement and high-probability counterclockwise rotation of occlusal plane, and counterclockwise rotation of mandible.
- Gummy smile with overdeveloped upper anterior and lower posterior teeth: Intrusion of upper anterior and lower posterior teeth, counterclockwise rotation of occlusal plane and mandible.
- No gummy smile with open bite anterior teeth and vertically overdeveloped upper and lower posterior teeth: Intrusion of upper and lower posterior teeth, clockwise rotation of occlusal plane, and counterclockwise rotation of mandible.
- Gummy smile with open bite anterior teeth and vertical overdevelopment of upper and lower posterior teeth: Intrusion of upper anterior, upper and lower posterior teeth, upward movement of occlusal plane, and counterclockwise rotation of mandible.
- Gummy smile with vertically overdeveloped upper and lower teeth: Intrusion of upper and lower anterior and posterior teeth, upward and high-probability counterclockwise rotation of occlusal plane, and counterclockwise rotation of mandible.
- No gummy smile with normal upper arch and vertically overdeveloped lower arch: Intrusion of lower anterior and posterior teeth, unchanged occlusal plane, and counterclockwise rotation of mandible.

Each combination targets specific characteristics of facial deformity, ensuring a tailored treatment approach to achieve optimal outcomes.

## A combination of orthognathic and orthodontic treatment strategies for protrusive facial deformities

In cases of skeletal malocclusions where patients have reached skeletal maturity or require treatment beyond orthodontic compensation, a combined orthognathic and orthodontic approach is frequently recommended.^111^ This comprehensive method integrates orthognathic surgery to correct jaw positions for optimal soft tissue esthetics, alongside pre- and postoperative orthodontic interventions. These interventions play a pivotal role in reconstructing the occlusal relationship between the upper and lower dental arches and reinstating the functionality of the stomatognathic system. This coordinated approach ensures not only esthetic improvement but also functional restoration, resulting in more favorable treatment outcomes.^112^

### Surgical design

Crafting a surgical plan for orthognathic surgery requires a thorough analysis of the morphological mechanisms and the severity of the malocclusion.^113^ In cases of severe protrusive facial deformities, it’s crucial to conduct a thorough assessment encompassing a precise understanding of the abnormal relationships and degrees of malformation within the dental arches and jaw positions in various dimensions and the dimensions of the upper airway.^114^ Such a systematic approach enables optimal orthodontic tooth movement and jaw displacement during orthognathic surgery, achieving ideal functional and esthetic outcomes.^115,116^

For patients with protrusive facial deformities, it’s important to be cautious of intraoperative mandibular advancement and traction on the masseter and suprahyoid muscle groups, as these maneuvers may heighten the risk of postoperative relapse.^117^ Research indicates that combining maxillary setback with mandibular advancement surgery yields superior postoperative stability compared to mandibular advancement surgery alone.^117^ Recognizing the constraints of orthognathic surgery is vital in treatment planning. It’s essential to consider the anatomical limitations of bone block mobility and the restrictions imposed by soft tissues. If refining local skeletal contours cannot be accomplished in a single procedure, additional surgeries may be necessary for further contouring. Additionally, there’s a growing trend in integrating orthognathic surgery with facial plastic surgery in craniofacial esthetic procedures. This integration offers the opportunity to incorporate interventions that modify nasal structure and facial soft tissue contours, further enhancing overall facial harmony and esthetic outcomes.^118^

### Maxillary osteotomy

#### Le Fort I osteotomy

**Fig. 4:**
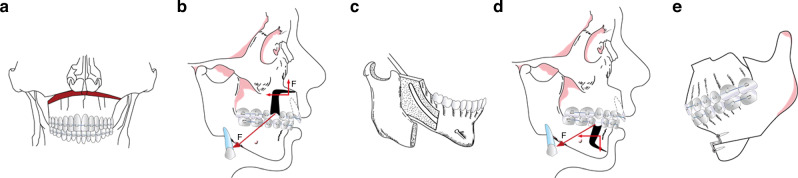
Schematic diagram of surgical design. **a** Le Fort I osteotomy. **b** AMO. **c** SSRO. **d** AMSO. **e** Genioplasty

The Le Fort I osteotomy (Fig. 4a) is a surgical procedure characterized by a horizontal bone cut positioned above the inferior margin of the pterygoid process, the anterior wall of the maxillary sinus, the zygomaticomaxillary buttress, and above the maxillary tuberosity.^119,120^ This technique mimics the anatomical course of a classic Le Fort Type I fracture. Its objective is to address maxillary hyperplasia by detaching and globally mobilizing the maxillary bone, encompassing the entire dental arch.^121^

#### Anterior maxillary osteotomy (AMO)

The AMO procedure (Fig. 4b) entails two key steps: initially, a horizontal bone cut is made above the inferior margin of the pterygoid process and the anterior wall of the maxillary sinus. This is followed by a vertical bone cut through the gap left post-extraction of the anterior molars.^122^ This sequential process results in the detachment of the bone segment in the anterior maxillary region. By mobilizing this bone segment, which includes the anterior nasal spine and the anterior floor of the nose, AMO effectively corrects protrusive facial deformities present in the anterior maxillary teeth and alveolar bone.^123^

### Mandibular osteotomy

#### Sagittal split ramus osteotomy (SSRO)

The SSRO procedure (Fig. 4c) involves a horizontal split of the mandible along its anatomical structure,^120,124,125^ as outlined in various references. This division divides the mandible into two segments: a proximal segment comprising the condyle, coronoid process, and mandibular angle, and a distal segment encompassing the body of the mandible, the entire mandibular dentition, and the neurovascular bundle of the inferior alveolar nerve. Through mobilization of the distal segment, SSRO effectively addresses mandibular protrusive facial deformities.

#### Anterior mandibular subapical osteotomy (AMSO)

The AMSO procedure (Fig. 4d) entails a sequential process. Initially, a horizontal bone cut is made at least 5 mm below the apices of the mandibular anterior teeth on the labial side of the mandible. Subsequently, a vertical bone cut is performed through the gap left post-extraction of the anterior molars.^126^ This sequential action results in the detachment of the bone segment in the mandibular anterior region. By mobilizing this bone segment, which includes the mandibular anterior teeth, AMSO effectively corrects protrusive facial deformities present in the anterior mandibular teeth and alveolar bone.^127^

#### Genioplasty

Genioplasty (Fig. 4e) is a surgical procedure that involves a horizontal cut below the apices of the mandibular anterior teeth and beneath the mental foramen. This incision allows for the mobilization of the chin bone segment. Through this process, genioplasty effectively corrects protrusive facial deformities in chin development.^128,129^

### Surgery-first orthognathic approach

The surgery-first approach (SFA) involves performing orthognathic surgery before initiating orthodontic treatment. The advantages of SFA include the immediate improvement of facial esthetics and a reduction in the duration of orthodontic treatment.^129^ The latter is related to a more physiological position of the teeth, the arrangement of the dental arch through surgery, and the regional acceleratory phenomenon after surgery. Cases with protrusive deformities that do not need too much preoperative orthodontic alignment and decompensation are regarded as indications of SFA.^130^ However, as with any immature technique, consensus regarding indications and surgical planning, as well as the evidence of long-term stability, is still lacking.^131–133^

### Preoperative and postoperative orthodontic treatment in orthognathic surgery

Preoperative orthodontics plays a critical role in preparing patients for orthognathic surgery by addressing several key objectives. Its primary focus includes eliminating compensatory tooth tilting, harmonizing the morphology and size of the maxillary and mandibular dental arches, facilitating jaw displacement, occlusal alignment, and the establishment of a stable occlusion post-surgery.^116,130^

Postoperative orthodontics further enhances treatment outcomes by refining tooth alignment and optimizing occlusal relationships, significantly contributing to the long-term stability of combined orthodontic and orthognathic treatments.^131,132^ Close coordination between orthodontic and orthognathic surgery specialist teams is paramount for achieving optimal results, with orthodontic treatment being an integral component of the overall therapeutic approach.

## Conclusions and perspectives

The protrusive deformity is one of the main causes affecting facial esthetics. Due to the complex etiology and diverse manifestations of protrusive deformities, orthodontic diagnosis and treatment strategies require consideration of multiple dimensions and comprehensive factors. Multidimensionality refers to the addition of a temporal dimension to the traditional three-dimensional concept, including sagittal, vertical, horizontal, and growth and development dimensions. Comprehensive factors encompass seven aspects, including teeth alignment, jaw relationship, facial contour, periodontal condition, upper airway patency, temporomandibular joints, and muscle balance. This consensus also provides a detailed discussion on the indications, intrusion strategies, and risk control associated with vertical control techniques for protrusive facial deformities.

While numerous clinical studies on the treatment of protrusive facial deformities exist, there is a future need for large-sample, multi-center randomized controlled clinical trials. There is a particular lack of prospective research on vertical control techniques, and evidence-based thinking needs to be integrated into the evaluation of treatment efficacy and postoperative stability analysis for protrusive deformities. With the continuous innovation of non-bracket invisible orthodontic technology and its combined application with other orthodontic techniques, the range of orthodontic appliance options for protrusion deformities is continuously expanding, benefiting more patients. In recent years, artificial intelligence has been gradually popularized in the medical field, and its powerful data analysis and processing capabilities have brought about significant changes in the diagnosis, treatment, and prognosis prediction of protrusive deformities.

In conclusion, the diagnosis and treatment of protrusive facial deformities are a systematic endeavor. As our understanding of the pathogenesis of protrusive facial deformities deepens, as clinical research continues to evolve, and as better research methods are applied in clinical practice, it will undoubtedly bring about more optimized results for the treatment and long-term stability of patients with protrusive facial deformities.
